# High Rates of Atrial Fibrillation among Inpatients with Heart Failure or Ischemic Stroke in Southwestern Uganda

**DOI:** 10.5334/aogh.5389

**Published:** 2026-10-29

**Authors:** Peter K. Olds, Precious Twesigye, Lydia Nakawesa, Ivan Kasomba, Jessica E. Haberer, Abeya Fardous

**Affiliations:** 1 Massachusetts General Hospital, Center for Global Health, Harvard Medical School, Boston, MA, USA; 2 Department of Medicine, Mbarara University of Science and Technology, Mbarara, Uganda; 3 Mbarara Regional Referral Hospital, Mbarara, Uganda

**Keywords:** cardiology, screening, arrhythmias, sub-Saharan Africa

## Abstract

*Background:* Atrial fibrillation (AF) affects over 37 million people worldwide, and its prevalence is projected to increase by 60% by 2050. Addressing AF is critical in Uganda and elsewhere in sub-Saharan Africa, where data are lacking despite the high prevalence of AF risk factors, comorbidities, and sequelae.

*Objectives:* To evaluate AF prevalence among inpatients with acute heart failure or acute ischemic stroke in Uganda.

*Methods:* Between November 4, 2024 and November 30, 2025, we recruited a cohort of inpatients at the Regional Referral Hospital in Mbarara, Uganda with either acute heart failure or acute ischemic stroke. We collected baseline clinical data and hospital outcomes, performed a transthoracic echocardiogram, and equipped participants with a 24-hour Holter monitor. A multivariable logistic regression model was fitted for a primary outcome of AF detection.

*Results:* Among 250 individuals, mean age was 61.7 years (SD 20.5), of whom 175 (70.0%) were women. 123 (49.2%) had acute heart failure; 127 (50.8%) had acute ischemic stroke. Holter monitors detected AF in 105 (42.0%), with a mean AF duration of 11.0 hours (IQR 1.0–21.9) with a bimodal distribution. Mean stroke risk (CHA_2_DS_2_VA) was 3.5 (SD 1.6). Factors associated with AF included increasing age in years (OR 1.04, 95% CI 1.02–1.06) and left atrial volume index (mL/m^2^) (OR 1.02, 95% CI 1.01–1.03).

*Conclusions:* We found high rates of AF among inpatients with acute heart failure or acute ischemic stroke, comparable to studies from the United States and Europe. These findings highlight the need to address AF in Uganda, with improved screening using long-term monitors, updated treatment protocols, and broadened access to cardiac care.

## Introduction

Atrial fibrillation (AF) is a cardiac arrhythmia that affects an estimated 37.6 million people worldwide and, due to aging populations and increasing risk factors like hypertension and diabetes, its prevalence is projected to increase by more than 60% by 2050 [1]. AF is associated with an increased risk of heart failure, myocardial infarction, ischemic stroke, dementia, and chronic kidney disease (CKD), accounting for an estimated 6 million disability-adjusted life years globally [2]. Addressing AF is critical in sub-Saharan Africa (SSA), and especially in Uganda, where there is a paucity of data despite high rates of both AF risk factors, comorbidities, and sequelae [3, 4].

Despite the high global burden, relatively few studies have examined the prevalence of AF in SSA, and those published to date have employed limited diagnostic measures. Studies looking at population-level prevalence in rural areas, including one in southwestern Uganda, have found rates of AF below 1% [5–7]. The only urban population-level study was done in Ethiopia and found a prevalence of 4.3% among adults over 40, which is closer to the estimated 4.5% prevalence among adults in the United States [8, 9]. Importantly, the population studies in SSA used 12-lead electrocardiogram (ECG), potentially missing paroxysmal cases.

Even fewer studies have looked at AF prevalence among high-risk patients in SSA, like those with ischemic stroke and heart failure, and none have been conducted in Uganda. Meta-analyses from studies in SSA have found prevalences of AF among patients with acute ischemic stroke and heart failure to be between 6.1% and 34.8% [10, 11] and between 5.0% and 31.0%, respectively. For example, the large, multinational THESUS-HF registry that included countries in SSA, including Uganda, showed an AF prevalence of 20.8%, though it was limited to patients with valvular heart failure [12]. Globally, the large EPOCH trial in the United States found an AF prevalence of 36.9% among inpatients with acute heart failure, and the REPORT-HF registry, which included patients hospitalized for acute heart failure from 44 countries (only South Africa from SSA), found an AF prevalence of 39% [13, 14]. For acute ischemic stroke, a national registry in Sweden found a prevalence of AF in 33.4% of patients, and a population-level study in France found a prevalence of 31.3% [15, 16].

Given the limited availability of prevalence data on AF in SSA and Uganda specifically, as well as the lack of long-term diagnostics and attention to high-risk patients, we conducted a prospective study of adults who were admitted to a regional referral hospital in southwestern Uganda with a diagnosis of acute heart failure or ischemic stroke and screened for AF using 24-hour Holter monitoring.

## Methods

### Study setting and population

This study took place at Mbarara Regional Referral Hospital (MRRH), a publicly operated tertiary care hospital in Mbarara, Uganda. MRRH is a 600-bed teaching hospital affiliated with Mbarara University of Science and Technology that serves the southwestern region of Uganda with a catchment area of 6–8 million people, offering both medical and surgical subspecialty services. The hospital admits approximately 220 heart failure and 140 new ischemic stroke adult patients each year [17].

### Sampling and recruitment

This study involved adults admitted to MRRH with a diagnosis of acute heart failure or new ischemic stroke. We recruited patients in conjunction with the hospital staff, who used admission registries to purposively identify potential participants with a similar ratio of men and women and a balanced distribution among participants with heart failure and stroke. Inclusion criteria included an established physician-made diagnosis of heart failure or ischemic stroke found on a computed tomography scan, being 18 years or older, and being able to speak either English or one of the local languages (Runyankole or Rukiga). Exclusion criteria included being pregnant (as pregnancy-related disease was considered beyond the scope of the current study), end-stage liver or kidney disease (as they could complicate heart failure diagnosis in a resource-limited setting), and an inability to provide informed consent, including impairment from intoxication or psychosis. All participants provided written consent for participation. Based on a population proportion of 20% in the THESUS-EF trial (as the largest trial done in SSA), we calculated that a sample size of 246 would provide prevalence estimates with a confidence level of 95% and a margin of error of 5%.

### Data collection

Upon enrollment, a research assistant administered a survey collecting sociodemographics (i.e., age, sex, district of residence, occupation, level of education, and tobacco and alcohol use) and comorbid conditions (i.e., history of cardiovascular, renal, and lung diseases, stroke, heart failure, malignancy, and human immunodeficiency virus [HIV]); performed a 12-lead ECG; and collected clinical data from the patient’s medical chart. A trained phlebotomist from MRRH took blood samples from all patient participants for basic metabolic panels and complete blood counts, which were subsequently analyzed at the MRRH clinical laboratory. Additional clinically ordered labs that resulted within 1 month of recruitment were extracted from the patient’s chart, including thyroid-stimulating hormone (TSH), lipid panel, aminotransferases, and CD4 cell count. A physician (author PT) performed a focused cardiovascular and neurological physical exam and transthoracic echocardiogram (TTE) on all participants. Finally, hospital outcomes and medications administered during hospital admission were recorded.

A research assistant equipped patients with a 24-hour Holter monitor (Norav Medical, Delray Beach, FL), placing three leads on participants using disposable foam electrodes. The research assistant collected the Holter monitor after 24 hours and uploaded the collected data to an encrypted server. Results were evaluated by a physician (author AF).

### Data analysis

Participant characteristics were summarized descriptively. Comparisons were made using one-sided ANOVA tests and Pearson’s chi-square tests for continuous variables and categorical variables, respectively.

The presence and duration of AF were determined using Holter data interpretation software (Norav Medical, Delray Beach, FL). ECGs were read by a physician (author AF) for the presence of AF. We made comparisons in rates of AF between heart failure and ischemic stroke participants using one-sided ANOVA tests and Pearson’s chi-square tests for continuous variables and categorical variables, respectively. A histogram of AF burden, calculated as duration in minutes, was made to visually demonstrate the prevalence of paroxysmal AF cases. CHA_2_DS_2_VA score was calculated for all patients, and scores were disaggregated by gender [18]. We calculated rates of anticoagulation therapy for patients identified with AF, and the 2024 European Society of Cardiology Guidelines for AF management were used as reference [19].

Logistic regression models were used to determine factors associated with the outcome of AF found on Holter monitoring. Univariable regression models were used to determine crude estimates for each exposure variable. Exposure variables included age, sex, medical comorbidities (including HIV serostatus), laboratory values, TTE findings, and clinical severity. Valvular disease was defined as history of rheumatic heart disease (RHD), history of valvular disease, or findings of aortic or mitral stenosis on TTE. Past medical history of heart failure was defined as either prior diagnosis of heart failure or presenting with acute heart failure, and past medical history of stroke was defined as either prior diagnosis of stroke or presenting with acute stroke. A multivariable regression model was fitted with *a priori* exposure variables (i.e., age, sex, histories of diabetes and hypertension, and left ventricular ejection fraction), in addition to those that were found to have a *P*-value of <0.1 on univariable analysis. We evaluated disease severity using two clinical severity scores (Sequential Organ Failure Assessment [SOFA] [20] and the Charlson Comorbidity Index [CCI] [21]). Potential interaction between variables based on clinical reasoning was explored. Variables in the regression model were evaluated for collinearity using uncentered variance inflation factor (VIF). A VIF score of 10 was used as a cut-off, with CCI meeting criteria. Variables with more than 10% missing data were not included in the regression. All data were analyzed using Stata SE 17.0 (StataCorp LLC, College Station, TX).

## Ethical Approval

Potential participants were approached in person for written informed consent. This study received approval through the Mbarara University of Science and Technology Research Ethics Committee (MUST-2023-1185), the Uganda National Council for Science and Technology (registration number HS3976ES), and the Mass General Brigham Institutional Review Board (2023P003413). Patients or the public were not involved in the design, conduct, reporting, or dissemination plans of this research. Due to the pilot nature of this study, formal patient and public involvement was not feasible during the initial development phase. However, the research team acknowledges this limitation. We intend to share the findings with relevant patient advocacy groups to inform the design of a larger, follow-up definitive trial, where a dedicated advisory group will be established from the outset.

## Results

Between November 4, 2024, and November 30, 2025, we screened 305 individuals (Figure 1), of whom 41 did not meet inclusion criteria and 14 declined study participation, resulting in a recruitment of 250 participants who were admitted to MRRH with a diagnosis of either heart failure or ischemic stroke (Table 1). The mean age was 61.7 years (standard deviation [SD] 20.5), and 175 (70.0%) were women. Just under half of patients (123; 49.2%) were admitted with acute heart failure, while 127 (50.8%) were admitted with acute ischemic stroke (Table 2). The most common medical comorbidity in past medical history was hypertension in 116 participants (46.4%), followed by heart failure in 104 (41.6%), diabetes in 40 (16.0%), and prior stroke in 38 (15.2%). The mean CCI was 3.8 (SD 1.9) and was significantly lower in participants with acute heart failure than those with ischemic stroke (3.4 vs. 4.1, *P* = 0.004). Length of stay was 5.9 days (SD 4.4) and was significantly higher in participants with acute heart failure than those with ischemic stroke (6.6 vs. 5.2, *P* = 0.001). Mean left ventricular ejection fraction was 50.6% (SD 21.1) and was significantly lower in those with acute heart failure than those with ischemic stroke (41.0% vs. 60.0%, *P* < 0.001). The in-hospital mortality for the cohort was 14 (5.6%).

**Figure 1:**
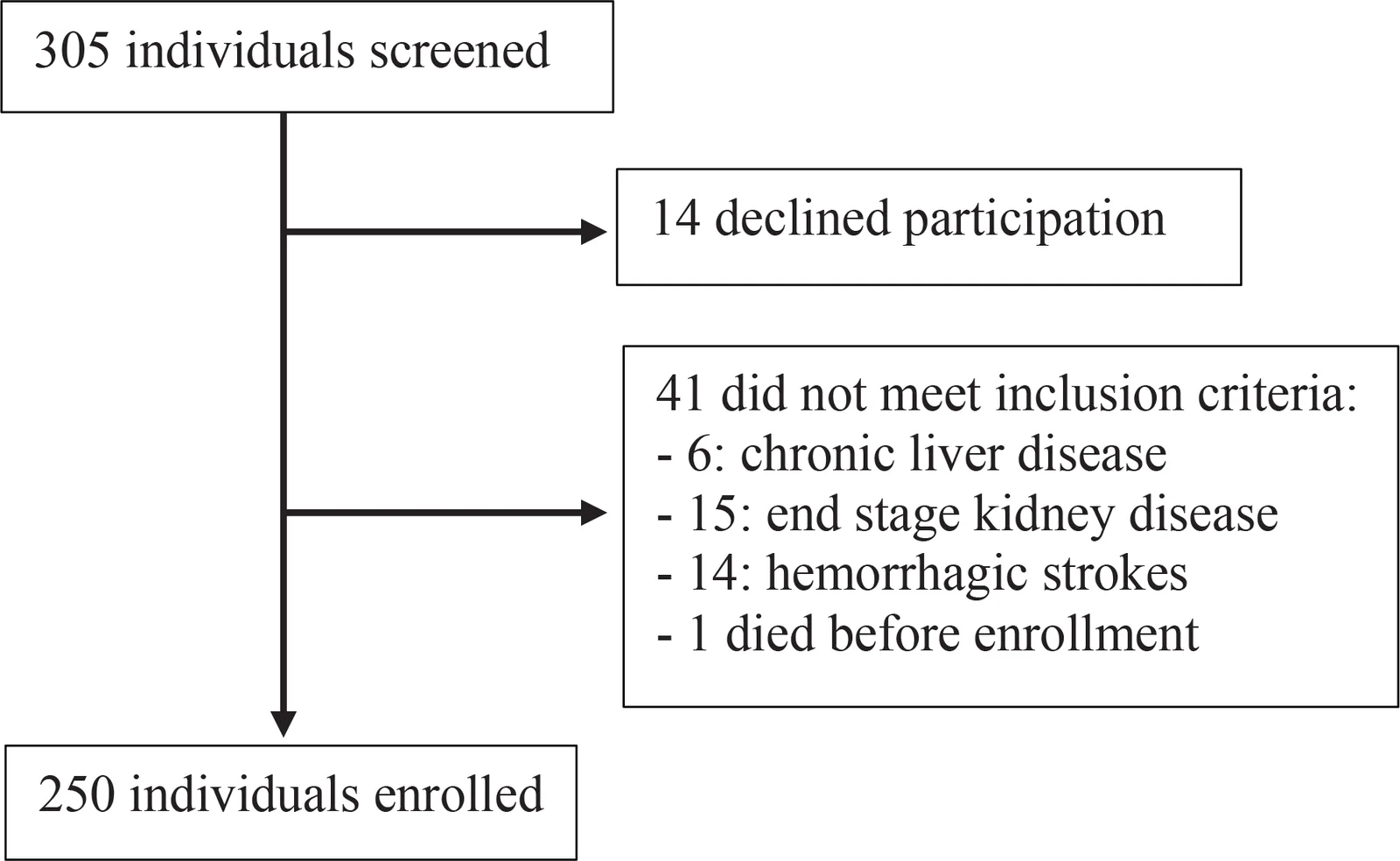
Recruitment flow diagram.

**Table 1:** Patient demographics and clinical characteristics. Demographic and clinical characteristics of study participants.

	ALL	HEART FAILURE	ISCHEMIC STROKE	*P*-VALUE
**Total (%)**	250	123	127	—
**Age, mean (SD)**	61.7 (20.5)	56.0 (20.6)	67.2 (18.9)	**<0.001**
**Female, *n* (%)**	175 (70.0)	83 (67.5)	92 (72.4)	0.39
**Past medical history (%)**				
Congenital heart disease	4 (1.6)	2 (1.6)	2 (1.6)	0.97
COPD/asthma	16 (6.4)	14 (11.4)	2 (1.6)	**0.002**
Diabetes	40 (16.0)	15 (12.2)	25 (19.7)	0.11
Prior heart failure	104 (41.6)	87 (70.7)	17 (13.4)	**<0.001**
HIV	12 (4.8)	4 (3.3)	8 (6.3)	0.26
Hypertension	116 (46.4)	50 (40.7)	66 (52.0)	0.07
Kidney disease	13 (5.2)	9 (7.3)	4 (3.2)	0.14
Liver disease	4 (1.6)	4 (3.3)	0 (0.0)	**0.04**
Myocardial infarction	7 (2.8)	4 (3.3)	3 (2.4)	0.67
Rheumatic heart disease	8 (3.2)	6 (4.9)	2 (1.6)	0.14
Tuberculosis	2 (0.8)	2 (1.6)	0 (0.0)	0.15
Prior stroke	38 (15.2)	0 (0.0)	8 (6.3)	**0.01**
**Social History (any current or prior) (%)**				
Alcohol use	41 (16.4)	22 (17.9)	19 (15.0)	0.53
Smoking	27 (10.8)	12 (9.8)	15 (11.8)	0.60
**Family History (%)**				
Cardiomyopathy	63 (25.2)	29 (23.6)	34 (26.8)	0.56
Sudden death	55 (22.0)	28 (22.8)	27 (21.3)	0.77
Coronary artery disease	17 (6.8)	9 (7.3)	8 (6.3)	0.75
**CCI, mean (SD)**	3.8 (1.9)	3.4 (1.8)	4.1 (1.9)	**0.004**
**SOFA on admission, mean (SD)**	1.5 (1.4)	1.3 (1.6)	1.7 (1.2)	**<0.001**
**Length of stay, mean (SD)**	5.9 (4.4)	6.6 (4.5)	5.2 (4.2)	**0.001**
**Ejection fraction, mean (SD)**	50.6 (21.1)	41.0 (20.8)	60.0 (21.1)	**<0.001**
**In-hospital mortality (%)**	14 (5.6)	9 (7.3)	5 (3.9)	0.25

SD: standard deviation; COPD: chronic obstructive pulmonary disease; HIV: human immunodeficiency virus; CCI: Charlson Comorbidity Index; SOFA: Sequential Organ Failure Assessment.

**Table 2:** Variables associated with the detection of atrial fibrillation (AF). Univariable and multivariable logistic regression findings of variables associated with AF.

	UNIVARIABLE		MULTIVARIABLE	
	OR (95% CI)	*P* VALUE	OR (95% CI)	*P* VALUE
**Age, years** (per year)	1.03 (1.01–1.04)	**<0.001**	1.04 (1.02–1.06)	**<0.001**
**Female sex** (reference: male sex)	1.8 (1.0–3.2)	**0.04**	1.8 (0.9–3.5)	0.10
**CCI** (per unit of score)	1.4 (1.2–1.6)	**<0.001**		
**SOFA** (per unit of score)	1.0 (0.8–1.2)	0.78		
**Past Medical History** (reference: no history)				
**Hypertension**	2.0 (1.2–3.3)	**0.01**	1.6 (0.8–3.1)	0.16
**Diabetes mellitus**	0.6 (0.3–1.3)	0.19	0.5 (0.2–1.2)	0.14
**Heart failure**	2.0 (1.2–3.4)	**0.01**	1.4 (0.6–3.2)	0.41
**Kidney disease**	0.9 (0.3–2.7)	0.79		
**Stroke**	0.8 (0.6–1.1)	0.14		
**HIV**	0.4 (0.1–1.7)	0.23		
**COPD/asthma**	1.8 (0.7–5.1)	0.24		
**Myocardial infarction**	0.5 (0.1–2.9)	0.47		
**Laboratory values**				
**eGFR** (per unit)	1.002 (0.997–1.007)	0.40		
**Hemoglobin** (per unit)	1.0 (1.0–1.1)	0.56		
**ECG findings** (reference: not present)				
**Left bundle branch block**	0.5 (0.2–1.3)	0.14		
**Right bundle ranch block**	2.6 (0.9–8.1)	**0.09**	1.0 (0.3–3.6)	0.97
**Transthoracic echocardiogram**				
**Ejection fraction, %** (per %)	1.0 (1.0–1.0)	0.94	1.01 (1.00–1.03)	0.15
**LA vol index** (per mL/m^2^)	1.02 (1.01–1.02)	**<0.001**	1.02 (1.01–1.03)	**<0.001**
**RA vol index** (per mL/m^2^)	1.02 (1.01–1.03)	**<0.001**	1.01 (1.00–1.02)	0.10
**Severe MR** (reference: no severe MR)	2.2 (1.1–4.1)	**0.02**	1.2 (0.5–2.9)	0.70
**Valvular disease** (reference: no valvular disease)	2.0 (0.8–5.2)	0.15		

OR: odds ratio; CI: confidence interval; CCI: Charlson Comorbidity Index; SOFA: Sequential Organ Failure Assessment; COPD: chronic obstructive pulmonary disease; HIV: human immunodeficiency virus; eGFR: estimated glomerular filtration rate; ECG: electrocardiogram; LA: left atrium; RA: right atrium; MR: mitral regurgitation.

Holter monitor detected AF in 105 participants (42.0%) (Figure 2). ECG detected 42 (16.8%) participants with AF, all of whom had AF detected on Holter monitor. Thus, ECG detected 42 of the 105 cases of AF in this cohort (40.0%). Of the 105 participants with AF detected on Holter monitoring, the CHA_2_DS_2_VA scores followed a normal distribution (Figure 3). Ninety-two of those with AF (87.6%) had a CHA_2_DS_2_VA score ≥2, meeting the recommended threshold for anticoagulation. Of those meeting criteria for anticoagulation, 35 (35.4%) were prescribed anticoagulation during hospitalization (Figure 2).

**Figure 2:**
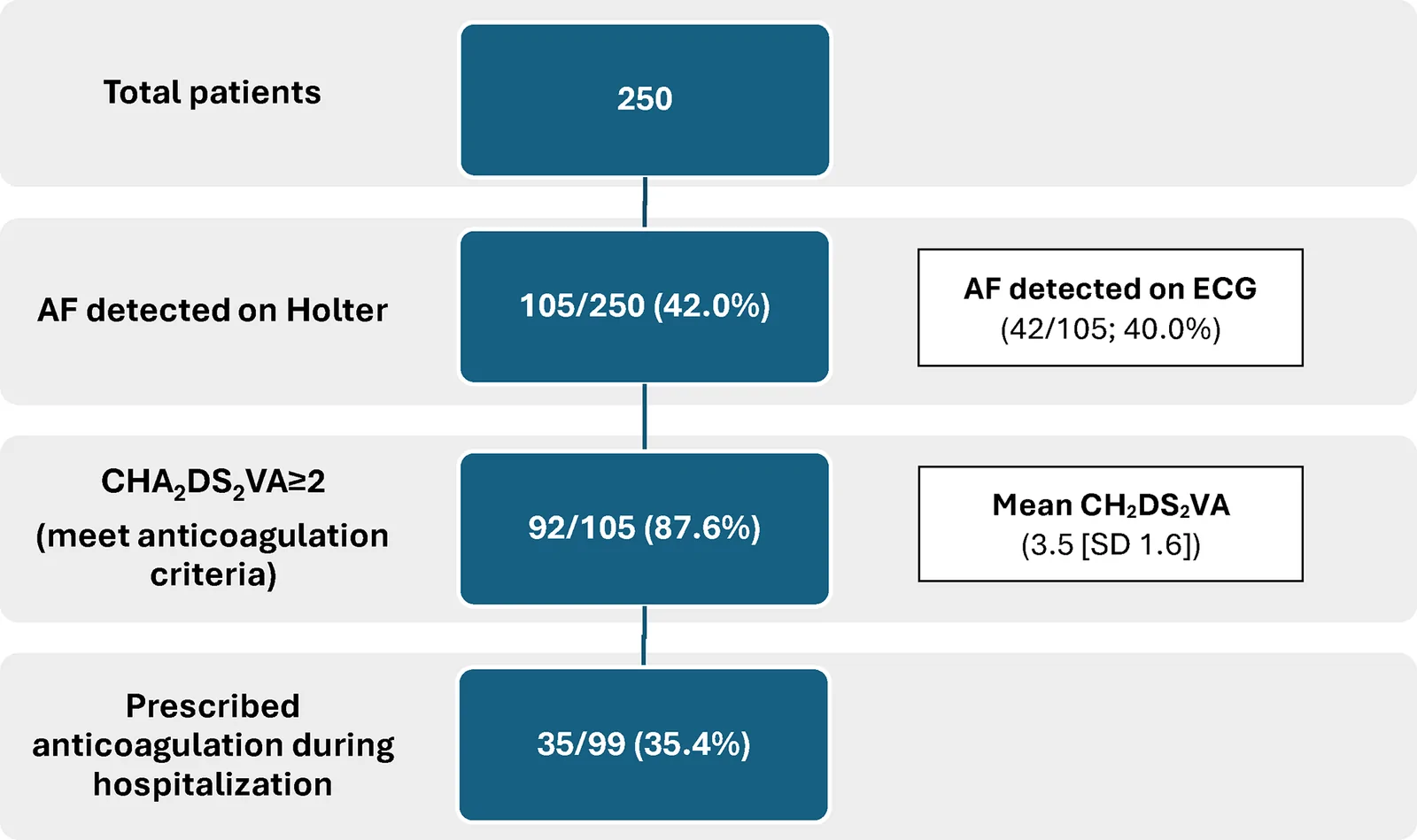
Atrial fibrillation (AF) detection and anticoagulation flowchart. Of note, all participants with AF on ECG had AF detected on the Holter monitor as well.

**Figure 3:**
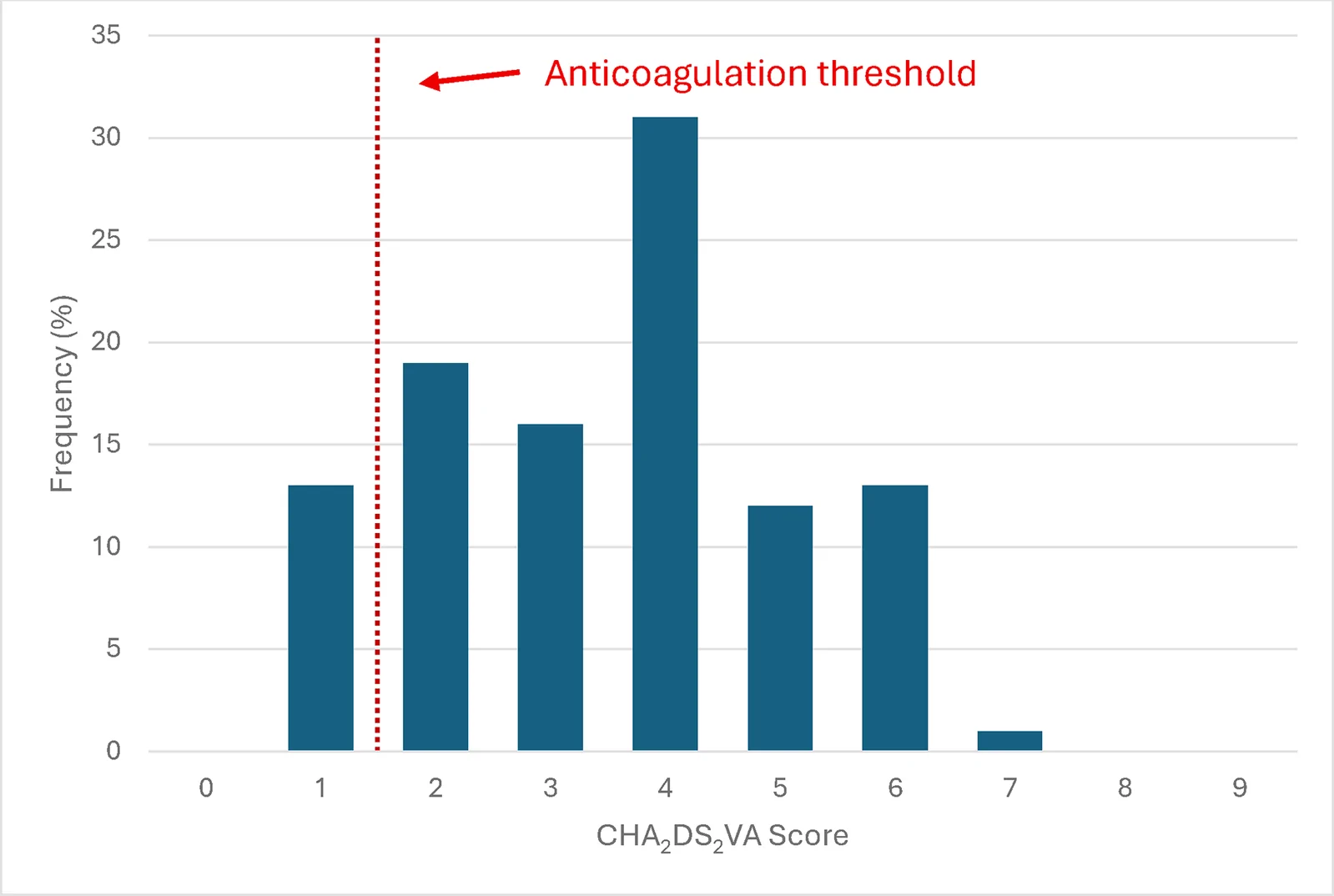
CHA_2_DS_2_VA distribution among those with documented AF by Holter monitoring. Recommended threshold for anticoagulation, a CHA_2_DS_2_VA score ≥2 is denoted by the dotted red line.

Over the 24 hours of monitoring, the mean burden of AF detected on Holter monitoring was 11.0 hours (interquartile range 1.0–21.9) (Figure 4). The distribution was bimodal, with 27 participants (25.7%) with less than 1 hour and 20 participants (19.0%) with more than 23 hours of AF time during the 24-hour monitoring.

**Figure 4:**
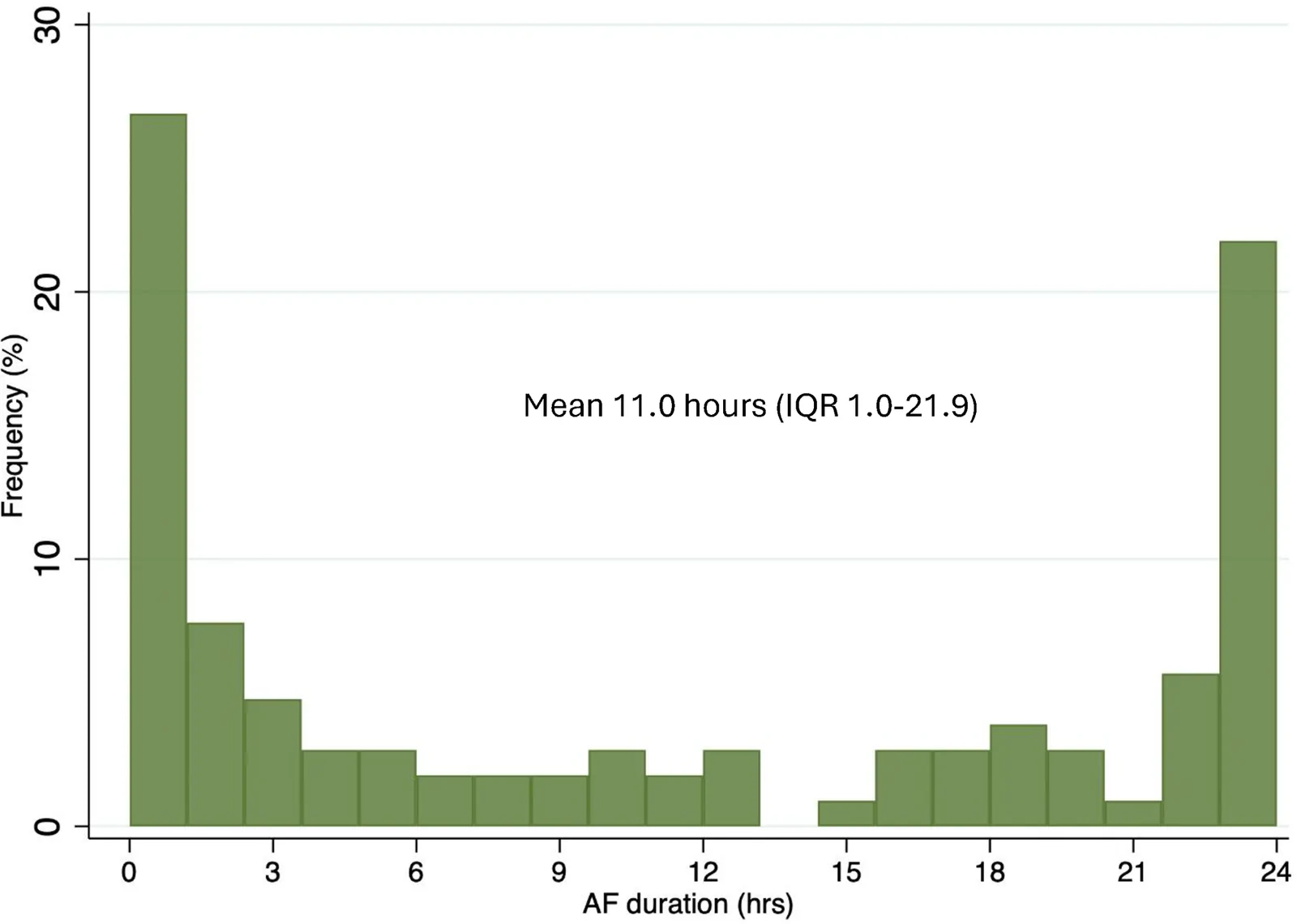
Atrial fibrillation (AF) burden in hours. Burden of AF detected by Holter monitor over 24 hours of monitoring.

In univariable analysis, factors positively associated with AF detection on the Holter monitor included age in years (odds ratio [OR] 1.03, 95% confidence interval [CI] 1.01–1.04), female sex (OR 1.8, 95% CI 1.0–3.2), CCI (OR 1.4, 95% CI 1.2–1.6), past medical history of hypertension (OR 2.0, 95% CI 1.2–3.3) or heart failure (OR 2.0, 95% CI 1.2–3.4), a right bundle branch block on ECG (OR 2.6, 95% CI 0.9–8.1), left atrial volume index per mL/m^2^ (OR 1.02, 95% CI 1.01–1.02), right atrial volume index per mL/m^2^ (OR 1.02, 95% CI 1.01–1.03), and severe mitral regurgitation (OR 2.2, 95% CI 1.1–4.1). In multivariable analysis, factors positively associated with AF detection on Holter monitor included age in years (OR 1.04, 95% CI 1.02–1.06) and left atrial volume index per mL/m^2^ (OR 1.02, 95% CI 1.01–1.03).

## Discussion

In this study evaluating AF burden among high-risk inpatients with heart failure or ischemic stroke, we found high rates of AF at 42.0%, with a mean AF burden of 11.0 hours over a 24-hour period. The distribution of AF duration was bimodal, with 25.7% of participants at less than an hour and 19.0% at over 23 hours. ECG screening detected 40% of AF cases. Most patients with AF were at high risk for ischemic stroke (CHA_2_DS_2_VA score ≥2 in 87.6% of AF patients), with only a minority treated with anticoagulation during hospitalization (35.4%). Factors positively associated with AF detection in our cohort included advancing age, and left atrium (LA) volume index on TTE.

The prevalence of AF in our cohort (42.0%) was higher than that reported in previous studies conducted among similar populations in SSA, with reported rates ranged from 5% to 34%. Our higher prevalence of AF is likely due to finding paroxysmal cases with the use of 24-hour Holter monitoring, given that ECG alone missed 60% of patients with AF in this cohort. The prevalence in this study is more in line with studies done in the Global North, where Holter monitoring or telemetry are standard of care, and where AF among patients with acute heart failure and acute ischemic stroke is between 30% and 39% [13–16]. This finding highlights that cases of AF in SSA are likely significantly more common than previously felt and potentially on par with rates in the Global North.

Significant risk factors for AF in our cohort included advancing age and LA volume on TTE, which are consistent with prior studies [22, 23]. LA volume is not only a risk factor for AF but also an independent predictor of AF severity, stroke, and AF recurrence [24–26]. While TTE is not readily available in Uganda and much of SSA, using point-of-care ultrasound (POCUS) to evaluate LA enlargement is a more feasible approach for evaluating patients in resource-limited settings [27]. As an example, in a study in Uganda for patients with RHD, Okello et al found that AF was associated with an LA diameter >40 mm [28]. While we did not measure LA diameter in this study, future work should look at using POCUS measurements for LA enlargement in non-RHD patients and correlations with disease severity in SSA.

Although we included the demographic AF risk factors, such as hypertension, diabetes, and CKD, in our multivariable regression, most did not meet significance in our study. This lack of significance may be because patients in Uganda often have incomplete past medical histories due to health system limitations, or may be a result of our high-risk population where rates of comorbidities were higher than population averages. Additionally, our population was focused only on those with acute heart failure or ischemic stroke. The lack of significance of acute heart failure and ischemic stroke in the multivariable regression may have been due to study design, rather than a lack of a true association. Of the few studies evaluating AF and its risk factors in Uganda, Okello et al. found AF to be the third most common complication among patients newly diagnosed with RHD in Mbarara [29], and a descriptive analysis by Lugero et al. of hospitalized patients in Kampala with AF found that heart failure was the most common comorbid condition encountered, in addition to high rates of embolic stroke at 12.8% [30].

Importantly, the primary risk factors for AF are increasingly common in Uganda and throughout the Global South [31]. The prevalence of non-communicable diseases (NCDs) that predispose patients to AF is rising across SSA, driven by the transition to westernized diets and sedentary lifestyles, an aging population, and likely increased detection rates [32, 33]. In Uganda, hypertension, diabetes, and CKD were estimated at 26%, 1.4%, and 6.8%, respectively, in 2016 and are estimated to be rising rapidly, likely due to increased detection and demographic changes. For example, the Global Burden of Disease Study in 2021 projects the age-standardized prevalence of type 2 diabetes to increase by more than 100% from 2021 to 2050 in East Africa [34–36]. Health facilities in SSA often lack the necessary tools to adequately diagnose and treat these NCDs, let alone have reliable telemetry or Holter monitors for screening high-risk patients for AF [10, 35–39]. Additionally, longitudinal outpatient care for managing chronic NCDs and their complications faces significant obstacles in SSA and remains unavailable to the majority of patients [40–43]. As life expectancies rise and demographic shifts continue in Uganda and throughout SSA, the number of patients with AF is likely to increase and put further strain on already fragile health systems.

A higher prevalence of AF and a growing number of at-risk individuals highlights the need to better screen for AF in Uganda and throughout SSA. We need more studies using long-term monitoring—rather than just ECG screening—to evaluate AF prevalence among both high-risk patients and the general population to better understand the true burden of disease and prioritize treatment. We can leverage novel mobile and wearable technologies for screening in resource-poor settings where there is limited availability of Holter and other forms of long-term monitoring. Our group has recently shown that smart watches are a feasible method of detecting AF among patients admitted with acute ischemic stroke and acute heart failure (Olds et al., submitted for publication). Additionally, given the prevalence of smartphones throughout SSA, smartphone-based screening, especially augmented with artificial intelligence, has significant potential to improve AF screening [44, 45].

The high CHA_2_DS_2_VA scores among our cohort not only highlight the significant multimorbidity of our population but also raise critical questions around anticoagulation therapy, especially because only a minority of our participants received anticoagulation while hospitalized. Warfarin remains the least expensive anticoagulant option for patients, but health systems in Uganda and throughout SSA often lack the ability to reliably monitor the international normalized ratio (INR) values needed to guide dosing. Direct oral anticoagulants (DOACs) have a more favorable bleeding profile and do not require monitoring, but price remains a major barrier for most patients. Additionally, the unreliable availability of reversal agents, blood bank products, CT scanning, endoscopy suites, and neurosurgical services make the question around who to treat with anticoagulation therapy, and at what CHA_2_DS_2_VA thresholds, incredibly difficult.

*Normative guidance on anticoagulation in SSA is lacking, and additional factors must be considered, like distance from a properly resourced facility and patient ability to access the medications, either due to cost or ineffective supply chains. Addressing system-level challenges through health system strengthening, like clinician training, improved referral pathways, and broader availability of diagnostic tools, will be essential in improving care for AF as well as other cardiovascular diseases. Additionally, the global cardiology community, ministries of health, and large foundations like the Clinton Health Access Initiative and Gates Foundation should call for more affordable DOAC prices to increase availability to patients throughout the Global South. Efforts like Stop TB’s Global Drug Facility and GAVI, the Vaccine Alliance, have proven successful in driving down costs for TB medications and vaccines and are blueprints for how the global cardiology community can broaden access to anticoagulation* [46, 47].

The major strength of this work is that it is the first study, to our knowledge, to prospectively screen for AF in SSA by systematically using long-term cardiac monitoring. Another strength was performing a TTE on all patients, providing granular imaging data unavailable in other AF studies in SSA. We also focused predominantly on high-risk patients, making this research more applicable in a clinical environment like Uganda, where one must maximize limited resources and find those who are most likely to benefit from anticoagulation and other interventions.

A notable weakness of this study is that it involved one tertiary hospital in Uganda, limiting its generalizability. We recruited a relatively small sample size and focused on high-risk patients rather than a population-based approach or one that included all hospitalized patients. Given hospital resource limitations and poor access to healthcare for many Ugandans, we had limited laboratory tests at our disposal, and participants likely had other comorbidities than stated in their charts.

## Conclusions

This study found high rates of AF among high-risk inpatients at a referral hospital in Uganda. Rates of AF were higher than those found in prior studies in SSA for the same population and were on par with rates found in the Global North. This study is the first screening for AF in SSA using 24-hour Holter monitoring and highlights the significant limitations of using ECG for AF screening. Future work should look at long-term monitoring for population-based screening. Finally, this study shows the critical need to address the cascade of care around cardiac care and anticoagulation therapy in SSA. Through concerted efforts and health system strengthening endeavors, we, as a global community, should work to improve NCD care delivery to address AF risk factors, bring more affordable DOAC therapy to our patients, and deliver system-level improvements to support patients during bleeding diatheses and other acute complications of AF.

## Data Sharing Statement

The datasets used and/or analyzed during the current study have been published in the Harvard Dataverse (https://doi.org/10.7910/DVN/EKWRCO).
